# More health workers needed for universal health coverage

**DOI:** 10.2471/BLT.18.021118

**Published:** 2018-11-01

## Abstract

Despite efforts to boost its health workforce, Bangladesh is struggling to make progress towards universal coverage of health services. Sophie Cousins reports.

On a stifling afternoon at Kunipara slum in Dhaka, the capital of Bangladesh, a group of women sit cross-legged on the floor of a health centre.

The women, who live in tin-roofed shacks perched above a canal, are all between seven and nine months pregnant, and are at a delivery centre run by BRAC, a Bangladesh-based international development organization, to prepare for giving birth.

Only around 37% of women in Bangladesh give birth in a health facility, fewer than one third have four antenatal care visits and only 6% receive the World Health Organization (WHO)-recommended eight visits or more. In addition, only 35.9% of deliveries are attended by skilled birth attendants.

Bangladesh has seen significant improvements in public health since gaining independence in 1971, notably achieving millennium development goal 4 by reducing child deaths by two-thirds ahead of 2015 target, and increasing the survival of women in pregnancy and childbirth and expanding immunization coverage.

Despite these health gains, the country is struggling to make overall progress due to the lack of qualified staff in the public health sector.

“Human resource management is our number one problem,” says Dr Mahbub Chowdhury, head of universal health coverage at the Health Systems Division of the icddr,b (formerly the International Centre for Diarrhoeal Disease Research, Bangladesh or ICDDR, B).

Chowdhury points to the lack of physicians, nurses and midwives as a core concern, with just 83 workers in those categories per 100 000 in 2018, which is nevertheless up from a reported 49 per 100 000 in 2008. Also of concern is the concentration of trained health-care providers, particularly physicians, in cities, leaving people in rural areas to generally make do with unqualified or semi-qualified health-care providers.

It has been estimated that less than 20% of health workers serve over 70% people living in rural areas. According to a recent paper published in the *Lancet* entitled *Harnessing pluralism for better health in Bangladesh*, there are as few as 11 doctors per 100 000 population in rural areas, compared to 182 per 100 000 in urban areas.

The government has developed several policies to address these issues. For example, to increase deployment and retention of health workers in rural areas, graduates from public medical schools are required to sign up to a minimum of two years of rural service. To improve retention, 20% of places in public medical schools are allocated to students from rural areas, who are more likely to return to their home areas.

While many countries face challenges in filling positions in remote rural areas, Bangladesh is struggling to fill positions in all settings.

“Human resource management is our number one problem.”

Dr Mahbub Chowdhury

“There are always about 20% vacancies among public sector health service posts,” says Dr Syed Masud Ahmed, professor and director of the Centre of Excellence for Health Systems and Universal Health Coverage at BRAC University in Dhaka.

According to a WHO report released this year, *The decade for health workforce strengthening in the South East Asian Region 2015–2024*, vacancies for nurses stand at 21%, for doctors at 34%, and for midwives at 60%.

Several factors drive these trends. The first, and most evident, is low levels of remuneration for health workers in the public sector.

Ahmed believes that despite recent efforts to increase public sector pay, low remuneration remains a significant brake on recruitment and retention.

Ahmed also points to other factors, such as the lack of transparency in decision-making on the posting, transfer and promotion of health workers, in addition to the tough work and living conditions they face, particularly in rural areas.

Once health workers are trained, many choose to go overseas or to find lucrative work in the private sector. Doctors working in the public sector are permitted to supplement their income with private practice (so-called dual practice) which often leads to neglect of patients in the public facilities. Bangladesh’s private health sector, predominantly staffed by public sector doctors and specialists, has grown considerably in the past 15 years and is largely unregulated.

As long as these health workforce issues go untackled, the public sector will continue to be starved of vital human resources. “If we continue in the same way, it will take quite some time for Bangladesh to reach the WHO-recommended ratio of health-care workers to population,” Ahmed says, referring to a 2006 *World health report* reference to 228 physicians, nurses and midwives per 100 000 population to achieve a targeted 80% coverage rate for skilled birth attendance and child immunization.

Recruitment and retention of women in the workforce is a major challenge on all levels of care. While the proportion of women doctors has increased from 31% in 2012 to 38% in 2017, women constitute 94% of the nursing workforce.

Bangladesh is the only country in the WHO South-East Asian Region to have more doctors than nurses, and one of three countries in the region where the proportion of female doctors is increasing. For Dr Valeria De Oliveira Cruz, who leads the health systems development team at the WHO Country Office in Bangladesh, boosting the number of nurses is critical to improving access to quality health services.

“If the government wants to move towards universal health coverage, it needs to invest in nursing, which is not only good for the health system but also for women’s empowerment,” she says. This means investing in nurses’ education but also ensuring adequate pay and conditions, once they are qualified.

De Oliveira Cruz recognizes, however, that there are other barriers to women entering the workforce. For example, many women have family and community obligations that make them reluctant to work shifts or be transferred elsewhere in the country.

Investing in nurses is not only vital to ensuring the quality of health care for the country’s ageing population, who are facing different health challenges, including an increasing burden of noncommunicable diseases, but also to empower women, achieve gender equality and support economic growth, three sustainable development goal targets.

“The health system is one of the biggest employers in any country, and presents a huge opportunity for young women. Investment in nurses is also an investment in women.”

Women are also needed to bolster the country’s cadre of midwives. 

In 2010, the government made a commitment to train more midwives, first, by introducing a six-month advanced midwifery certificate and then, in 2012, with the introduction of a three-year diploma in midwifery.

However, a target to train 3000 midwives by 2015 was not met and, to date, only 1143 newly trained midwives have been deployed.

“Bangladesh has done a lot with limited resources, but it needs to invest far more in its health system, especially in the development of health-care workers who can deliver high quality services.”

Valeria De Oliveira Cruz

Dire shortages of midwives in Bangladesh are a major factor for maternal and newborn health outcomes. Only about a third of babies are delivered by skilled birth attendant. “More often than not babies are delivered at home by traditional birth attendants or family members who have no formal training,” says Dr Sohely Rahman, senior programme specialist at BRAC.

The lack of progress on building a health workforce that is fit for purpose in Bangladesh comes despite recent efforts to address the issue, including the Bangladesh Health Workforce Strategy, which charts workforce needs and project demands up to 2030.

“The 2015 strategy is aimed at achieving a skilled, motivated and responsive health workforce in adequate numbers that are available equitably across the country,” says Dr Michelle McIsaac, a health economist from the Health Workforce Department at WHO headquarters in Geneva.

The strategy initially concentrates on the public sector, comprising health workers under the Ministry of Health and Family Welfare. Key strategy focuses are developing and maintaining quality health workforce at all levels, and recruiting, deploying and retaining health workers.

Clearly the impact of such a comprehensive strategy takes time to be felt, but there are reasons to fear that without adequate funding, and the governance and stewardship required to ensure proper use of the resources raised, the initiative will fall short of expectations. According to the Bangladesh National Health Accounts for 2015, the government spends only 0.69% of GDP on health, making it one of the lowest spenders on health globally.

“With that level of funding it’s very difficult to get a well functioning health system,” says De Oliveira Cruz. “Bangladesh has done a lot with limited resources, but it needs to invest more in its health system, especially in the development of health workers who can deliver high quality services.” The kind of services being provided at the BRAC delivery centre where Hridi (not her real name), one of the pregnant mothers at the centre, plans to have her baby.

Hridi delivered her first child at home, assisted by family members. Luckily, the delivery went well, and she and her baby survived. This time, she is leaving nothing to chance, and will get the guidance and care she needs. “A health facility birth I will have,” she says, smiling broadly. “And then I won’t have any more children.”

**Figure:**
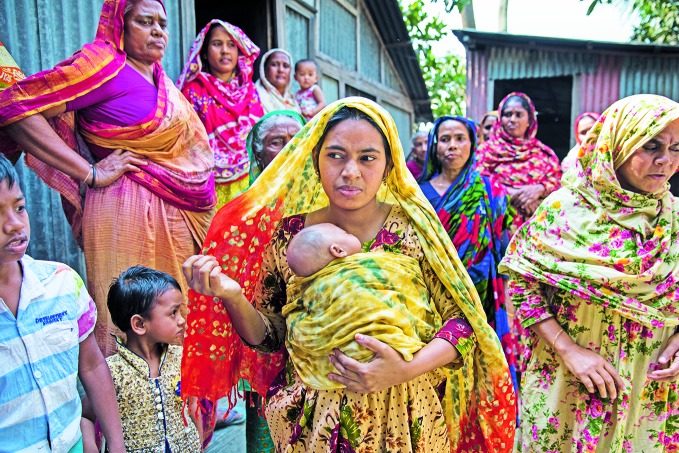
Mother uses kangaroo technique to hold her 8-week-old baby in Dhaka, Bangladesh

© UNICEF/UN0198371/Njiokiktjien VII Photo

**Figure:**
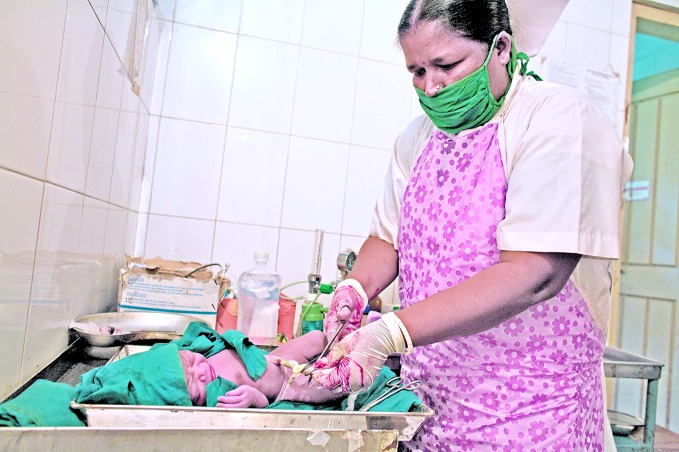
Midwife attends to newborn baby in Bangladesh

© UNICEF/UNI7903/Nooran

